# Superlattices of Gadolinium and Bismuth Based Thallium Dichalcogenides as Potential Magnetic Topological Insulators

**DOI:** 10.3390/nano13010038

**Published:** 2022-12-22

**Authors:** Alexandra Yu. Vyazovskaya, Evgeniy K. Petrov, Yury M. Koroteev, Mihovil Bosnar, Igor V. Silkin, Evgueni V. Chulkov, Mikhail M. Otrokov

**Affiliations:** 1 Laboratory of Nanostructured Surfaces and Coatings, Tomsk State University, Tomsk 634050, Russia; 2 Laboratory of Electronic and Spin Structure of Nanosystems, St. Petersburg State University, St. Petersburg 198504, Russia; 3 Institute of Strength Physics and Materials Science, Tomsk 634021, Russia; 4 Donostia International Physics Center (DIPC), 20018 Donostia-San Sebastián, Basque Country, Spain; 5 Departamento de Polímeros y Materiales Avanzados: Física, Química y Tecnología, Facultad de Ciencias Químicas, Universidad del País Vasco UPV/EHU, 20080 Donostia-San Sebastián, Basque Country, Spain; 6 Centro de Física de Materiales (CFM-MPC), Centro Mixto CSIC-UPV/EHU, 20018 Donostia-San Sebastián, Basque Country, Spain; 7 IKERBASQUE, Basque Foundation for Science, 48011 Bilbao, Basque Country, Spain

**Keywords:** density functional theory, magnetic properties, electronic structure, topological insulator

## Abstract

Using relativistic spin-polarized density functional theory calculations we investigate magnetism, electronic structure and topology of the ternary thallium gadolinium dichalcogenides TlGd$Z_{2}$ (Z= Se and Te) as well as superlattices on their basis. We find TlGd$Z_{2}$ to have an antiferromagnetic exchange coupling both within and between the Gd layers, which leads to frustration and a complex magnetic structure. The electronic structure calculations reveal both TlGdSe${}_{2}$ and TlGdTe${}_{2}$ to be topologically trivial semiconductors. However, as we show further, a three-dimensional (3D) magnetic topological insulator (TI) state can potentially be achieved by constructing superlattices of the TlGd$Z_{2}$/(TlBi$Z_{2}$)${}_{n}$ type, in which structural units of TlGd$Z_{2}$ are alternated with those of the isomorphic TlBi$Z_{2}$ compounds, known to be non-magnetic 3D TIs. Our results suggest a new approach for achieving 3D magnetic TI phases in such superlattices which is applicable to a large family of thallium rare-earth dichalcogenides and is expected to yield a fertile and tunable playground for exotic topological physics.

## 1. Introduction

Magnetic topological insulators (MTIs) attract a great deal of attention nowadays [1,2,3,4,5,6,7,8,9] as their unique properties could find applications in dissipationless topological electronics [10], random-access memories [11], topological quantum computations [12], micro/nanoelectromechanical devices [13,14], and others. In particular, MTIs enable observation of such fundamental and practically-promising phenomena as quantum anomalous Hall [6,8,15,16] and topological magnetoelectric effects [15,17,18] (QAH and TME, respectively). Previously, transition metal elements doping [1,16] and magnetic proximity effect [19,20] approaches have been used in attempts to realize these phenomena in experiments. Although the QAHE [16,21,22] and TME [23,24,25] have been achieved, their observation has been limited to very low temperatures (≤2 K) due to the inherently disordered nature of the corresponding materials [26,27,28].

Intrinsic MTIs and heterostructures on their basis have emerged recently as a promising alternative to observe the above-mentioned quantized topological effects at elevated temperatures [2,3,29,30,31,32,33,34,35,36,37,38,39,40,41,42,43,44,45,46,47,48,49]. Discovery of the first representative of the MTI class, the antiferromagnetic (AFM) TI [50] MnBi${}_{2}$Te${}_{4}$ [2,34,35,36,37], has led to an appearance of an entire family of intrinsic MTIs, including (MnBi${}_{2}$Te${}_{4}$)·n(Bi${}_{2}$Te${}_{3}$) [42,44,45,51,52,53], MnBi${}_{2-x}$Sb${}_{x}$Te${}_{4}$ [41,54], (MnSb${}_{2}$Te${}_{4}$)·n(Sb${}_{2}$Te${}_{3}$) [31,54,55,56], Mn${}_{2}$(Bi,Sb)${}_{2}$Te${}_{5}$ [57,58,59], and MnBi${}_{2}$Se${}_{4}$ [31,33,60,61], which enabled significant advances. Indeed, thin MnBi${}_{2}$Te${}_{4}$ flakes have been found to show a Chern insulator state up to 30 K, achieved under an external magnetic field, but without Landau levels [5,6,62]. This has been followed by the realization of the axion insulator state [5], and eventually, of the QAHE (i.e., the QHE without external field) at 1.5 K [6], in accord with the theoretical predictions [34,35]. Very recently, the QAH regime has also been reported in the (MnBi${}_{2}$Te${}_{4}$)·n(Bi${}_{2}$Te${}_{3}$) heterostructures up to 6.5 K [8]. Noteworthy, apart from the MnBi${}_{2}$Te${}_{4}$-family, AFMTI state has also been predicted in some Eu-based compounds, such as EuIn${}_{2}$As${}_{2}$ [63,64,65].

To further consolidate the field of intrinsic MTIs, discovering new families of magnetic topologically-nontrivial systems is highly desirable. Indeed, different crystal structures and/or atomic compositions should lead to different magnetic properties, as compared to those of the MnBi${}_{2}$Te${}_{4}$-family, which should enable the realization of novel magnetic topological states of matter. In relation to a search of new MTI materials, it is instructive to observe that Mn(Bi,Sb)${}_{2}$Te${}_{4}$ and their homologous series are isostructural to those of the non-magnetic $A^{\mathrm{IV}}$(Bi,Sb)${}_{2}$Te${}_{4}$, where $A^{\mathrm{IV}}$= Ge, Sn, or Pb [66], which had been established to be 3D TIs before Mn(Bi,Sb)${}_{2}$Te${}_{4}$ were synthesized. Obviously, the similarity of the crystal structures and atomic compositions of the non-magnetic 3D TIs $A^{\mathrm{IV}}$(Bi,Sb)${}_{2}$Te${}_{4}$ to those of (back then) topologically uncharacterized compound MnBi${}_{2}$Te${}_{4}$ hints at a likelihood of topological non-triviality of the latter, which was later confirmed both theoretically and experimentally [2,34,35,36,37]. In the present study, we will be looking for the new 3D MTI systems by choosing as a starting point yet another family of the ternary non-magnetic 3D TIs, i.e., thallium pnictogen dichalcogenides Tl$XZ_{2}$, where X= Bi or Sb and Z= Se or Te [67,68,69,70].

**Figure 1:**
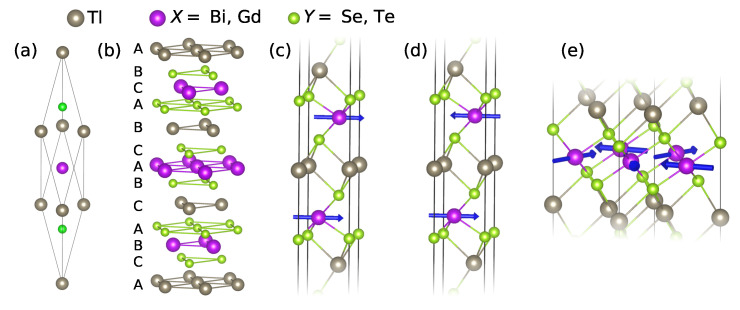
(**a**) Rhombohedral unit cell of Tl$XZ_{2}$ (X= Bi, Gd, and Z= Se, Te), with grey, purple, and green balls showing Tl, *X*, and *Z* atoms, respectively. (**b**) Sketch showing the hexagonal character of the atomic layers as well as the ABCABC stacking. (**c**,**d**) Interlayer FM (**c**) and AFM (**d**) alignment of the FM Gd layers. The blue arrows show the orientations of the magnetic moments. (**e**) Intralayer non-collinear 120${}^{\circ }$ AFM structure.

Tl$XZ_{2}$ adopt the α-NaFeO${}_{2}$ structure (space group $R\bar{3}m$), in which the hexagonal atomic layers form the *fcc*-type stacking sequence (ABCABC) with the Tl${}^{+}$ and $X^{3+}$ cation layers separated by the $Z^{2-}$ anion layers, –Tl–*Z*–*X*–*Z*–Tl–*Z*–*X*–*Z*–, see Figure 1a,b. In contrast to the aforementioned Mn(Bi,Sb)${}_{2}$Te${}_{4}$ or $A^{\mathrm{IV}}$(Bi,Sb)${}_{2}$Te${}_{4}$ families, that are van der Waals layered materials made of septuple layer blocks, Tl$XZ_{2}$ have no well-defined structural blocks and, thus, no van der Waals bonding, which endows the family with more 3D-like properties. Following the above-outlined logic, we may wonder if the Tl$XZ_{2}$ family includes magnetic members and, if it does, whether they are topologically non-trivial or not. In fact, compounds with the α-NaFeO${}_{2}$ structure and Tl$XZ_{2}$ formula, in which the $X^{3+}$ cation position is occupied by Gd instead of Sb or Bi, were synthesized in the powder form quite some time ago [71,72,73,74]. However, in spite of that, their magnetic and electronic properties remain poorly characterized. Indeed, on the experimental side, there only have been hints of a complex helicoidal spin arrangement in TlGdSe${}_{2}$ [75]. On the theoretical side, the available density functional theory (DFT) studies of TlGd$Z_{2}$ [76] take neither spin–orbit coupling (SOC) nor Hubbard *U* into account, which are very important for the compounds of this family, containing both heavy elements and strongly correlated 4*f* electrons. As for a possible topologically non-trivial state in TlGd$Z_{2}$, which might be suspected to exist, as it does in their non-magnetic pnictogen-based Tl$XZ_{2}$ TI counterparts, this question remains unanswered so far.

In this paper, we present a theoretical study of magnetism and electronic structure of TlGd$Z_{2}$ (Z= Se and Te) using relativistic spin-polarized ab initio calculations, taking the Hubbard *U* corrections into account. We find that for both TlGdSe${}_{2}$ and TlGdTe${}_{2}$ the intralayer non-collinear 120${}^{\circ }$ AFM state is energetically more favorable than the FM one, indicative of the AFM coupling within Gd layers. On the other hand, the interlayer exchange coupling is found to be AFM as well, although significantly weaker, which is due to a larger Gd-Gd separation along the *c* axis. The electronic structure calculations including SOC reveal both TlGd$Z_{2}$ compounds to be topologically-trivial semiconductors with band gaps of about 1.26 and 0.56 eV for Z= Se and Te, respectively. However, we show that constructing TlGdTe${}_{2}$/(TlBiTe${}_{2}$)${}_{n}$ (n=1,2) superlattices, based on the alternating –Tl–Te–Gd–Te– and –Tl–Te–Bi–Te– units, creates a desired SOC-driven bulk band gap inversion, making these systems potential 3D MTIs both intrinsically and in the forced FM state, which can be realized under an external magnetic field. Taking into account a great variety of the TlGd$Z_{2}$-like compounds, selenides, tellurides and even sulfides, containing instead of Gd other rare earth (RE) elements, such as Pr, Nd, Sm, Tb, Dy, Ho, Er, and Tm [71,72,77], our study uncovers a new large family of potential magnetic topological materials of the (TlRE$Z_{2}$)${}_{m}$/(Tl$XZ_{2}$)${}_{n}$ type (X= Bi, Sb and Z= S, Se, Te). In these systems, by varying RE, *X*, *Z*, and m,n-parameters, it should be possible to tune not only the exchange coupling (both intra- and interlayer), but also the magnetic anisotropy and, hence, the overall spin structure. Moreover, the electronic properties such as the fundamental band gap size and character (inverted or not), 4f levels position, as well as the SOC strength can be varied as well. Thus, the TlRE$Z_{2}$/Tl$XZ_{2}$ superlattices are expected to provide a fertile playground for the realization of new and exotic physics. In a broader sense, our results suggest an alternative approach for achieving a 3D MTI state. Namely, apart from doping non-magnetic TIs with transition metal elements or looking for a material combining magnetism and topology intrinsically, these properties can be realized in a superlattice of the isostructural stoichiometric magnetic trivial and non-magnetic topological insulators.

## 2. Calculation Details

The calculations were performed within the DFT using the projector augmented-wave method [78], implemented in VASP [79,80]. The generalized gradient approximation (GGA) was used to describe the exchange-correlation energy [81]. The Hamiltonian contained scalar-relativistic corrections and the SOC was taken into account by the second variation method [82]. We used the GGA + *U* approach [83,84] to describe the strongly localized Gd 4f states. The values U=6.7 eV and J=0.7 eV were used, typically yielding a reasonable description of the electronic structure of the Gd-containing compounds [85,86]. The electronic structures, calculated with VASP, were verified against those obtained within the full-potential linearized augmented plane wave (FLAPW) method [87] within the GGA+U approach [85,88] under the fully localized limit [89] as implemented in FLEUR [90] and a good agreement was found.

For the TlGd$Z_{2}$ systems, the full crystal structure optimization, i.e., that of the *a* and *c* lattice constants, c/a ratio, and atomic coordinates, was performed in the rhombohedral unit cells, while for the superlattices the hexagonal unit cells were used. The lattice parameters obtained for the bulk Tl$XZ_{2}$ (Table 1) are in good agreement with the experimental ones [71,72,73,75], being slightly larger than the latter, within 1.5 %. The intralayer exchange coupling was investigated using the $(\sqrt{3}\times \sqrt{3}R30^{\circ })\times 1$ hexagonal cells containing 3 atoms per layer, which is needed to model the intralayer non-collinear 120${}^{\circ }$ AFM structure. To study the interlayer exchange coupling, we used oblique cells having a hexagonal ab basis with the (1×1) in-plane periodicity and containing 8, 16, and 24 atoms in the cases of TlGd$Z_{2}$, TlGdTe${}_{2}$/(TlBiTe${}_{2}$)${}_{1}$, and TlGdTe${}_{2}$/(TlBiTe${}_{2}$)${}_{2}$, respectively. Atomic positions were relaxed using a force tolerance criterion of 10 meV/Å.

Surface band structures were calculated within a tight-binding approach based on the maximally localized Wannier functions [91,92] using iterative Green function method [93] implemented in WannierTools package [94].

$Z_{2}$ topological invariant $\nu_{0}$ was calculated according to [95]:

$${\left(-1\right)}^{\nu_{0}}=\prod_{i\in \Gamma_{TRIM}}\delta_{i},$$

where

$$\delta_{i}=\prod_{m=2,4,6...}^{N}\zeta_{m}\left(\Gamma_{i}\right).$$

$\zeta_{m}\left(\Gamma_{i}\right)$ are eigenvalues of wave function parity operator of occupied states with number *m* at time-reversal invariant momenta (TRIM) points $\Gamma_{TRIM}$. Calculations of $Z_{4}$ topological invariant were carried out according to Refs. [7,96] as

$$Z_{4}=\sum_{i\in \Gamma_{INV}}\frac{n{\left(\Gamma_{i}\right)}^{+}-n{\left(\Gamma_{i}\right)}^{-}}{2}.$$

where $n{\left(\Gamma_{i}\right)}^{\pm }$ is a number of occupied states with expectation values of parity ±1 at inversion symmetry invariant momenta points $\Gamma_{i}$.

## 3. Results and Discussion

### 3.1. Magnetic State of TlGd$Z_{2}$

TlGd$Z_{2}$ has a markedly anisotropic crystal structure, in which the distance between Gd atoms in the basal ab plane is significantly smaller than that along the *c*-axis. In such quasi-2D magnets, there is a hierarchy of exchange interactions: the intralayer couplings are usually significantly stronger than the interlayer ones. We scrutinize these interactions by calculating the total energies of the following three spin configurations. In the first two, the interaction between the nearest neighbors in the Gd layers is assumed to be ferromagnetic, while their alignment along the *c*-axis can be either FM (Figure 1c) or AFM (Figure 1d), enabling evaluation of the interlayer exchange coupling. Determination of the intralayer coupling requires consideration of an additional spin structure, that could occur if the interaction between the nearest neighbors in the Gd layers was antiferromagnetic. It is well-known that in combination with a 2D hexagonal lattice, AFM exchange coupling leads to a frustration [97], typically resolving itself into a non-collinear pattern, in which three spin sublattices form an angle of $120^{\circ }$ with respect to each other. Such a spin structure is shown in Figure 1e and will be hereinafter referred to as non-collinear antiferromagnetic (NCAFM).

While the character and strength of the interlayer exchange coupling can be conveniently characterized by $\Delta E_{⊥}=E_{AFM}-E_{FM}$, a simple difference between $E_{NCAFM}$ and either of $E_{AFM}$ and $E_{FM}$ does not describe the intralayer coupling precisely. Indeed, apart from a dominant contribution from the intralayer alignment, the total energies of the cases with FM Gd planes (i.e., $E_{AFM}$ and $E_{FM}$) also include a smaller one, coming from the interlayer exchange coupling, which is either purely AFM or FM. However, in the NCAFM case, there can be no purely AFM or FM interlayer alignment. This is because of the way this spin structure combines with the ABCABC stacking of atomic layers in TlGd$Z_{2}$ (Figure 1b,e), leading to frustration. In detail, a Gd magnetic moment from one layer, that has three nearest magnetic neighbors in each of the two adjacent Gd layers (Figure 1b), cannot simultaneously be either AFM- or FM-coupled to all of them because we necessarily assume all of the Gd layers to be NCAFM (which is to model the intralayer AFM interaction case). Therefore, both $E_{NCAFM}-E_{FM}$ and $E_{NCAFM}-E_{AFM}$ inevitably contain a certain contribution from the coupling between layers, since it does not cancel out. Indeed, the energetical favorableness of a particular interlayer configuration (FM or AFM) makes the corresponding difference smaller and, vice versa, its unfavorableness makes it larger, which, however, has nothing to do with the intralayer coupling since $E_{NCAFM}$ stays the same. Let us therefore account for this spurious contribution as follows: $\Delta E_{\left|\right|}=E_{NCAFM}-E_{FM}+\delta =E_{NCAFM}-E_{AFM}-\delta$, where δ eliminates the contribution from the interlayer coupling. By doing simple math, we get $\Delta E_{\left|\right|}=E_{NCAFM}-\left(E_{FM}+E_{AFM}\right)/2$ and $\delta =-\Delta E_{⊥}/2$, δ being positive (negative) if the AFM (FM) interlayer exchange coupling is energetically favorable.

According to our total-energy calculations, $\Delta E_{\left|\right|}<0$ for both TlGdSe${}_{2}$ and TlGdTe${}_{2}$. In the former case, the energy gain, $|\Delta E_{\left|\right|}|$, amounts to 9.5 meV per Gd pair, while in the latter it is 11.7 meV (Table 1). We thus can conclude that there is a pronounced tendency towards the non-collinear $120^{\circ }$ AFM structure formation in the Gd layers of the TlGd$Z_{2}$ compounds. On the other hand, our calculations show that $\Delta E_{⊥}$ is negative, too, and equal to −1.3 (−2.5) meV per Gd pair for TlGdSe${}_{2}$ (TlGdTe${}_{2}$), signaling the AFM character of the interaction between neighboring Gd planes. As expected, the interlayer coupling is significantly weaker than the intralayer one, which is due to the quasi-2D magnetic character of these compounds, determined by their layered crystal structure. Indeed, the interlayer Gd-Gd distance along *c* is significantly larger than the in-plane one, e.g., 8.13 Å vs. 4.24 Å for TlGdSe${}_{2}$. Still, these values cannot be considered as negligible, since $\Delta E_{⊥}$ of similar magnitude have been calculated for MnBi${}_{2}$Te${}_{4}$ and MnSb${}_{2}$Te${}_{4}$ compounds [34,45,55], that are known to be in the magnetically three-dimensional regime. The Gd local magnetic moments in both TlGdSe${}_{2}$ and TlGdTe${}_{2}$ are roughly equal to 7 $\mu_{B}$ (S=7/2), in a reasonable agreement with the available experimental data [98] and DFT calculations [76].

As has been discussed above, due to the combination of the intralayer NCAFM state and the ABCABC-type stacking of atomic planes, the interlayer exchange coupling leads to magnetic frustration. To get a deeper insight into the influence of the interlayer coupling on the TlGd$Z_{2}$ magnetic structure, different spin alignments between the $120^{\circ }$-ordered planes have been considered, as is further exemplified by TlGdTe${}_{2}$. We first note that in the NCAFM configuration considered each Gd moment has one ferromagnetically aligned nearest neighbor in each adjacent Gd layer, while the other two nearest neighbor moments are rotated by $\pm 120^{\circ }$ with respect to it. The presence of the FM-coupled pairs might seem energetically unfavorable since the interlayer interaction tends to align local moments antiferromagnetically because $\Delta E_{⊥}<0$. However, if we choose an antiparallel alignment in the same pairs, the total energy stays essentially the same (within the calculation accuracy). This is because the energy decrease due to the AFM alignment is compensated by the energy increase due to the interactions with the other two nearest neighbor moments, pointing at $\pm 60^{\circ }$. The sum of the latter two moments yields a vector of the same magnitude as the first one but points in the opposite direction.

Another possibility is that the interlayer configuration is helimagnetic, which is compatible with the interlayer magnetic frustration [99,100]. It would also be in line with the results of the electron paramagnetic resonance study of TlGdSe${}_{2}$ [75], pointing towards a possible helimagnetic state. To explore this, we have considered a spin structure in which the $120^{\circ }$ pattern of the Gd local moments rotates by an angle $\phi =30^{\circ }$ when going from one Gd layer to another along the *c*-axis. Here, each Gd moment is perpendicular to that of one of the nearest neighbors in each adjacent Gd layer, while with respect to the other two nearest neighbor moments it deviates by $30^{\circ }$ from the collinear FM and AFM alignments. In such a situation, no energy gain arises either, as compared to the NCAFM structure. Still, we can exclude neither a possibility of the helix angle being incommensurate with the hexagonal symmetry nor an appearance of a broken-helix magnetic order with more than one rotation angle [65]. Noteworthy, our magnetic anisotropy energy calculations show that the SOC does not favor any particular direction of the local magnetic moment, either in-plane or out-of-plane. In this situation, the role of the dipole-dipole interaction becomes crucial, which generally favors locking of the magnetic moments within the basal plane and realization of the tail-chase structure, although a canting towards the out-of-plane direction or the amplitude modulations can also occur in some cases [101,102,103]. It is thus evident that the exact 3D magnetic ground state structure of both TlGdTe${}_{2}$ and TlGdSe${}_{2}$ is very difficult to determine using the DFT calculations. The neutron diffraction measurements, which is a standard tool used to resolve magnetic structures in experiment [65,99], are desirable for TlGd$Z_{2}$.

### 3.2. Electronic Structure of TlGd$Z_{2}$  (Z= Se, Te)

**Figure 2:**
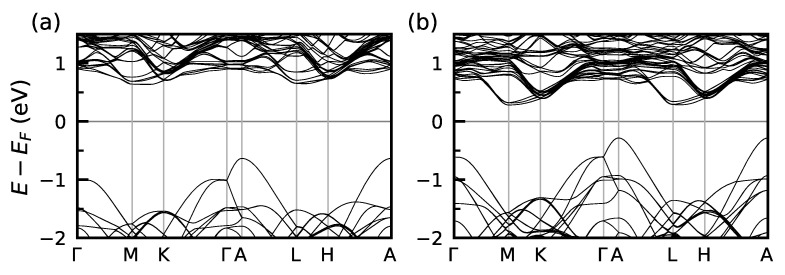
Bulk electronic structure of (**a**) TlGdSe${}_{2}$ and (**b**) TlGdTe${}_{2}$, calculated for the NCAFM spin alignment shown in Figure 1e.

The bulk band structures of TlGd$Z_{2}$ are presented in Figure 2 (the NCAFM structure, shown in Figure 1e, is assumed). It can be seen that both TlGdSe${}_{2}$ and TlGdTe${}_{2}$ show insulating spectra with indirect band gaps. According to the density of states calculations, the gap sizes are 1.26 eV and 0.56 eV, respectively. In both cases, the valence band maxima are formed by *p*-states of a chalcogen, while the conduction band minima are composed of the Tl *p*- and Gd *d*-states. No signature of the SOC-driven band gap inversion is observed from the analysis of the orbital composition of the states forming the band gap edges. We further confirm the absence of the inversion by direct calculations of the density of states performed at different values of the SOC constant. Thus, we can conclude that TlGd$Z_{2}$ (Z= Se, Te) are topologically trivial magnetic semiconductors.

### 3.3. TlGdTe${}_{2}$/(TlBiTe${}_{2}$)${}_{n}$  Superlattices

**Figure 3:**
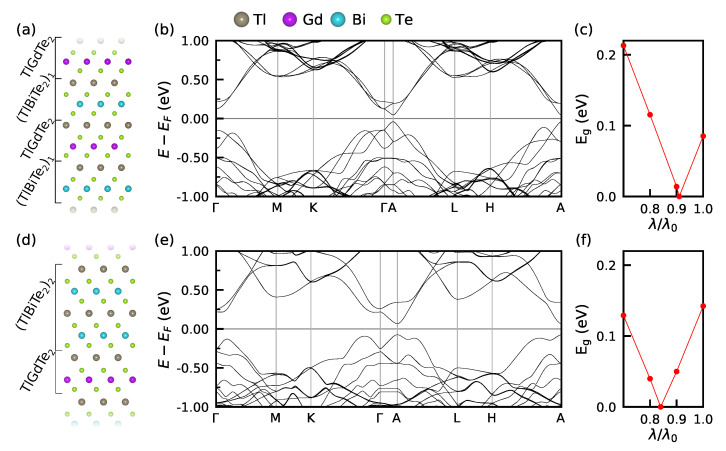
(**a**,**d**) Crystal structures of the TlGdTe${}_{2}$/(TlBiTe${}_{2}$)${}_{n}$ superlattices for n=1 (**a**) and n=2 (**d**). (**b**,**e**) Bulk electronic structure of the TlGdTe${}_{2}$/(TlBiTe${}_{2}$)${}_{n}$ superlattices for n=1 (**b**) and n=2 (**e**). Note that the number of bands is larger for the n=1 system due to a larger number of the atoms in the hexagonal unit cell: 24 for n=1 vs. 12 for n=2. This is because the ABCABC stacking dictates that the number of atoms in the hexagonal cell is divisible by three. (**c**,**f**) Evolution of the bulk gap size with the change of the SOC constant λ from its natural strength $\lambda_{0}$ to about 0.7$\lambda_{0}$. The NCAFM spin alignment in the Gd layers is assumed in the calculations.

Taking advantage of a relatively small lattice mismatch (Δa≈2%) between TlGdTe${}_{2}$ and the non-magnetic TI TlBiTe${}_{2}$ (Supplementary Figure S1), the similarity of their atomic composition, as well as their having the same $R\bar{3}m$ crystal structure (Figure 1a), we consider bulk superlattices of the TlGdTe${}_{2}$/(TlBiTe${}_{2}$)${}_{n}$ type with n=1 and 2. Their structure, shown in Figure 3a,d, comprises four-layer –Tl–Te–Gd–Te– units alternated with single (n=1) or double (n=2) four-layer unit of –Tl–Te–Bi–Te–. We have chosen TlGdTe${}_{2}$ as opposed to TlGdSe${}_{2}$ since the former has a smaller bulk band gap, which should be more easily inverted due to the strong SOC of Bi thus potentially allowing the realization of a topologically non-trivial magnetic insulating system.

After a full structural optimization (see lattice parameters in Table 2), the magnetism of TlGdTe${}_{2}$/(TlBiTe${}_{2}$)${}_{n}$ has been studied. On the one hand, we find that the intralayer NCAFM structure is by about 11.5 meV more favorable than the FM one for both n=1 and n=2, similar to pure TlGdTe${}_{2}$. On the other hand, the interlayer exchange coupling for n=1 weakens by an order of magnitude as compared to the latter ($\Delta E_{⊥}=-0.19$ meV [Table 2)] vs. −2.5 meV [Table 1)]), which is due to the increase of the Gd-Gd interlayer distance. When going from n=1 to n=2 it weakens by another order of magnitude.

We then study the electronic structure of the TlGdTe${}_{2}$/(TlBiTe${}_{2}$)${}_{n}$ superlattices, as previously assuming the NCAFM configuration. In Figure 3b,e, it is seen that, as compared to pure TlGdTe${}_{2}$, the superlattices have a direct band gap (located in the A-point) and the gap size is much smaller (cf. data in Table 1 and Table 2). At that, the gap of the n=2 system (141 meV), having a higher Bi content, is larger than that of n=1 (86 meV). The latter fact, along with the direct character of the band gap, points towards band gap inversion due to SOC, which is enhanced due to the presence of Bi. To confirm the band gap inversion we have calculated its value as a function of the SOC strength, λ. The results, presented in Figure 3c,f, show that at $\lambda /\lambda_{0}\approx 0.91$ ($\lambda /\lambda_{0}\approx 0.84$) the gap of the n=1 (n=2) system is closed, while at other values of $\lambda /\lambda_{0}$ it is non-zero, meaning that at $\lambda =\lambda_{0}$ it is indeed inverted around the A-point, which indicates a potential MTI state of both TlGdTe${}_{2}$/(TlBiTe${}_{2}$)${}_{n}$.

Since, similarly to pure TlGd$Z_{2}$, we do not know the exact magnetic ground state of TlGdTe${}_{2}$/(TlBiTe${}_{2}$)${}_{n}$ because of the coexistence of frustrated exchange interactions with dipolar contributions to magnetic anisotropy, we cannot reliably establish their topological classification. It should, however, be noted, that even for a complex magnetic structure, there may be a symmetry, which could give rise to a topological classification. For example, it has recently been found by neutron diffraction that EuIn${}_{2}$As${}_{2}$ exhibits a low-symmetry broken-helix antiferromagnetic order with two helix turn angles, which nevertheless supports a magnetic topological-crystalline axion insulator protected by the combination of a 180${}^{\circ }$ rotation and time-reversal symmetry [65].

Taking advantage of relatively weak exchange interactions in TlGdTe${}_{2}$/(TlBiTe${}_{2}$)${}_{n}$, it should in principle be possible to impose a ferromagnetically polarized state by applying an external magnetic field, as in the compounds of the MnBi${}_{2}$Te${}_{4}$ family [5,6,62]. In the artificial ferromagnetic state with a magnetization perpendicular to the Gd layers, the TlGdTe${}_{2}$/(TlBiTe${}_{2}$)${}_{n}$ are characterized by gapped spectra (not shown) with bulk band gaps of 69 and 131 meV for n=1 and 2, respectively. By varying the SOC constant λ we find that the band gaps are inverted, too. Moreover, the $Z_{4}$ invariant calculations show that both systems are axion insulators ($Z_{4}=2$), having the quantized topological magnetoelectric response in the bulk and chiral hinge modes [7,96]. Finally, the calculated $Z_{2}$ invariant shows that in the paramagnetic state these systems are time-reversal symmetric strong 3D TIs.

**Figure 4:**
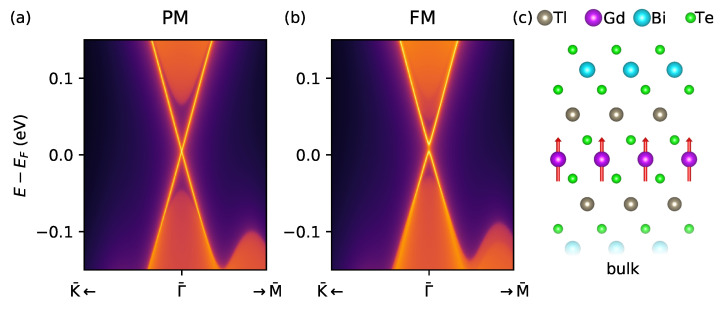
Surface electronic structure of TlGdTe${}_{2}$/(TlBiTe${}_{2}$)${}_{1}$, calculated for the PM state (**a**) and FM state (**b**) for one the Te termination shown in (**c**). The regions with a continuous spectrum correspond to the bulk projected states.

To further illustrate the topologically non-trivial character of our superlattices, in Figure 4 we show the surface electronic structure of ${\mathrm{TlGdTe}}_{2}/{\left({\mathrm{TlBiTe}}_{2}\right)}_{1}$ in the paramagnetic (PM) and ferromagnetic (FM) states. In the PM state, a linearly dispersing gapless surface state is clearly seen within the fundamental bulk band gap (Figure 4a), as expected for the time-reversal symmetric strong 3D TI. In contrast, when the system is driven in the FM state (which can be achieved by the out-of-plane external magnetic field), the Dirac point splits, consistently with what has been predicted for some compounds of the (Mn(Bi,Sb)${}_{2}$Te${}_{4})\cdot n$((Bi,Sb)${}_{2}$Te${}_{3}$) family in the FM state [55,104,105,106].

Finally, we would like to discuss the similarities and differences between the here discussed MTI superlattices TlGdTe${}_{2}$/(TlBiTe${}_{2}$)${}_{n}$ and intrinsic MTIs of the (MnBi${}_{2}$Te${}_{4}$)·n(Bi${}_{2}$Te${}_{3}$) and (MnSb${}_{2}$Te${}_{4}$)·n(Sb${}_{2}$Te${}_{3}$) families. Pretty much as the latter systems, composed of the building blocks of the magnetic (Mn(Bi,Sb)${}_{2}$Te${}_{4}$) and nonmagnetic ((Bi,Sb)${}_{2}$Te${}_{3}$) compounds, our superlattices are made of two stoichiometric systems, too. However, while (Mn(Bi,Sb)${}_{2}$Te${}_{4})\cdot n$((Bi,Sb)${}_{2}$Te${}_{3}$) are compounds themselves, TlGdTe${}_{2}$/(TlBiTe${}_{2}$)${}_{n}$ can hardly be considered as such. Indeed, (Mn(Bi,Sb)${}_{2}$Te${}_{4})\cdot n$((Bi,Sb)${}_{2}$Te${}_{3}$) can be grown in the bulk single crystal form, while applying the bulk growth strategy to the Tl(Gd,Bi)$Z_{2}$ system would, most likely, result in a disordered TlGd${}_{x}$Bi${}_{1-x}Z_{2}$ solid solution (found to be paramagnetic down to 2 K at x=0.1) [107]. In this sense, TlGdTe${}_{2}$/(TlBiTe${}_{2}$)${}_{n}$ are somewhat similar to delta-doped systems, such as digital magnetic alloys [108], representing transition metal monolayers embedded in a matrix of semiconductor material, studied previously in the field of diluted magnetic semiconductors. We propose that TlGdTe${}_{2}$/(TlBiTe${}_{2}$)${}_{n}$ can be synthesized using layer-by-layer growth techniques, such as molecular beam epitaxy or atomic layer deposition, that have proven successful for growing layered van der Waals and non-van der Waals materials, complex heterostructures and superlattices [32,56,109,110,111].

## 4. Conclusions

In summary, using ab initio and tight-binding calculations we have studied magnetism and electronic structure of the TlGd$Z_{2}$ (Z= Se, Te) compounds as well as TlGdTe${}_{2}$/(TlBiTe${}_{2}$)${}_{n}$ superlattices. Our results suggest a complex magnetic ground state in all these systems, which is due to the combination of the antiferromagnetic exchange interaction within Gd layers (resolving itself into the non-collinear spin structure), the fcc-type layer stacking, and the presence of the interlayer exchange coupling. As far as the electronic structure is concerned, TlGd$Z_{2}$ appear to be topologically-trivial semiconductors. However, a magnetic topological insulator state can be induced by constructing superlattices of TlGd$Z_{2}$ with isostructural Tl-based non-magnetic TIs. In this way, we predict that TlGdTe${}_{2}$/(TlBiTe${}_{2}$)${}_{n}$ potentially hosts a magnetic topological insulator state both intrinsically and under an external magnetic field. Our study not only suggests a new approach for achieving magnetic topological states of matter, but also uncovers a new large family of the TlRE$Z_{2}$/(Tl$XZ_{2}$)${}_{n}$ superlattices (RE = Pr, Nd, Sm, Gd, Tb, Dy, Ho, Er, and Tm; X= Bi, Sb; Z= S, Se, Te) with tunable magnetic, electronic and topological properties.

## Figures and Tables

**Table 1:** Optimized hexagonal lattice constants, *a* and *c* (Å), local magnetic moment on the Gd atom, *m*$\left(\mu_{B}\right)$, total energy differences of the AFM and FM configurations inside the Gd layer ($\Delta E_{\left|\right|}=E_{NCAFM}-\left(E_{FM}+E_{AFM}\right)/2$, meV/[Gd pair]) and between the neighboring layers ($\Delta E_{⊥}=E_{AFM}-E_{FM}$, meV/[Gd pair]), and the bulk band gap $E_{g}$ (eV).

Compound	*a*	*c*	*m*	$\Delta E_{\left|\right|}$	$\Delta E_{⊥}$	$E_{g}$
${\mathrm{TlGdSe}}_{2}$	4.24	23.25	7.00	−9.5	−1.3	1.26
${\mathrm{TlGdTe}}_{2}$	4.49	24.53	7.02	−11.7	−2.5	0.56

**Table 2:** The same as Table 1, but for TlGdTe${}_{2}$/(TlBiTe${}_{2}$)${}_{n}$. Note that for n=1, the *c* parameter is approximately twice as large as the one for n=2 because the hexagonal unit cell of the former contains 24 atoms as compared to 12 of the latter.

Superlattice	*a*	*c*	$\Delta E_{\left|\right|}$	$\Delta E_{⊥}$	$E_{g}$
${\mathrm{TlGdTe}}_{2}/{\left({\mathrm{TlBiTe}}_{2}\right)}_{1}$	4.54	48.46	−11.8	−0.19	0.086
${\mathrm{TlGdTe}}_{2}/{\left({\mathrm{TlBiTe}}_{2}\right)}_{2}$	4.56	24.02	−11.3	−0.02	0.141

## Data Availability

Not applicable.

## Supplementary Materials

The following are available online at https://www.mdpi.com/article/10.3390/nano13010038/s1, Figure S1: Comparison of the bulk electronic structures of TlBiTe${}_{2}$ and TlGdTe${}_{2}$.

## Abbreviations

The following abbreviations are used in this manuscript:

MTI	Magnetic topological insulator
FM	Ferromagnetic
AFM	Antiferromagnetic
NCAFM	Noncollinear antiferromagnetic
QAHE	quantum anomalous Hall effect
TME	Topological magnetoelectric effect
DFT	Density functional theory
SOC	Spin-orbit coupling
