# Supplementary material for: Superlattices of Gadolinium and Bismuth Based Thallium Dichalcogenides as Potential Magnetic Topological Insulators
Source: Nanomaterials (Basel). 2022 Dec 22;13(1):38. doi: 10.3390/nano13010038 (PMC9824305; doi:10.3390/nano13010038)
Supplement: Supplementary file 1 [file nanomaterials-13-00038-s001.zip › nanomaterials-2079813-supplementary.pdf]

Supplementary Materials: Superlattices of gadolinium and bismuth based thallium dichalcogenides as potential magnetic topological insulators

Alexandra Yu. Vyazovskaya, Evgeniy K. Petrov, Yury M. Koroteev, Mihovil Bosnar, Igor V. Silkin, Evgueni V. Chulkov, and Mikhail M. Otrokov

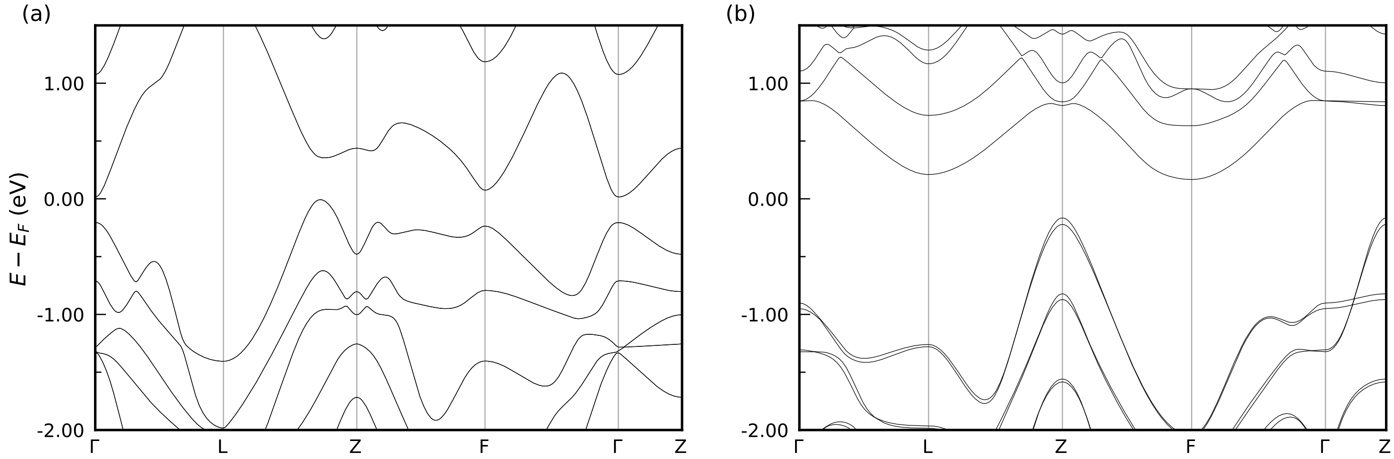

Figure S1. Bulk electronic structure of (**a**) $TlBiTe_2$ and (**b**) $TlGdTe_2$ in the rhombohedral 1x1x1 cell. For the latter compound, the calculation was performed for the forced ferromagnetic state with out-of-plane orientation of magnetic moments.

For $TlBiTe_2$, the Γ-point band gap is about 0.2 meV, while the one at the Z-point is roughly 1 eV. In turn, in $TlGdTe_2$ (irrespectively of the magnetic state) the band gap in the Γ-point is large, while the Z-point is close to that in $TlBiTe_2$. Hybridization of the $TlBiTe_2$ bands $TlGdTe_2$ in a superlattice (and folding along k_z in $TlBiTe_2/(TlGdTe_2)_1$) lead to appearance of the inversion at the A-point of the hexagonal Brillouin zone (Fig. 3, maintext). Recall, that the hexagonal cells in Fig. 3 of the maintext are dictated by the NCAFM structure.
